# Supplementary material for: The single nucleotide polymorphism rs1053230 modulates kynurenine 3-monooxygenase stability and is associated with cognitive and mood phenotypes
Source: bioRxiv. 2026 May 21:2026.05.19.726163. Preprint. [Version 1] doi: 10.64898/2026.05.19.726163 (PMC13228610; doi:10.64898/2026.05.19.726163)
Supplement: Supplement 1 [file media-1.docx]

**Supplemental Data File 1**

**The single nucleotide polymorphism rs1053230 modulates kynurenine 3-monooxygenase stability and is associated with cognitive and mood phenotypes**

Mary E W Collier^1^, Georgia Ceeney^2^, Joshua Chiappelli^3^, Sathyasaikumar Korrapati^3^, Thong Huy Cao^4,5,6,7^, Paulene Quinn^4,6^, Junfeng Ma^8^, Aashti Shauriq^1^, Nicolas Sylvius^9^, Edward J Hollox^1^, Donald J L Jones^6,10^, Andrew Hudson^2^, Elliot L Hong^11^, Nigel Scrutton^8^, Robert Schwarcz^3^, Flaviano Giorgini^1^

^1^ Division of Genetics and Genome Biology, University of Leicester, University Road, Leicester, LE1 7RH, UK

^2^ School of Chemistry, University of Leicester, University Road, Leicester, LE1 7RH, UK; Leicester Institute for Structural & Chemical Biology, Henry Wellcome Building, Lancaster Road, LE1 7HB.

^3^ Department of Psychiatry, Maryland Psychiatric Research Center, University of Maryland School of Medicine, Baltimore, Maryland, USA

^4^ Division of Cardiovascular Sciences, School of Medical Sciences, College of Life Sciences, University of Leicester, Leicester, LE1 7RH, United Kingdom

^5^National Institute for Health and Care Research Leicester Biomedical Research Centre, University Hospitals of Leicester NHS Trust, Glenfield Hospital, Leicester, LE3 9QP, United Kingdom

^6^Leicester van Geest Multi-OMICS facility, University of Leicester, Leicester, LE1 7RH, UK

^7^British Heart Foundation Centre of Research Excellence, College of Life Sciences, University of Leicester, Leicester, LE1 7RH, United Kingdom

^8^ Manchester Institute of Biotechnology, University of Manchester, Princess Street, Manchester, M1 7DN, UK

^9^ Genomics Core Facility, University of Leicester, Leicester, UK

^10^Leicester Cancer Research Centre, University Hospitals of Leicester NHS Trust, Leicester Royal Infirmary, University of Leicester, Leicester, LE1 5WW, United Kingdom

^11^ Department of Psychiatry and Behavioural Sciences, McGovern Medical School, University of Texas Health Science Center, Houston, Texas, USA


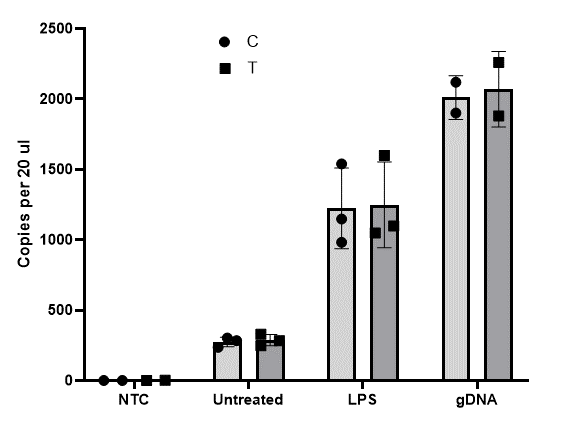


**Figure S1. rs2275163 genotype had no effect on KMO pre-mRNA stability.** THP-1 cells were treated with LPS (0.1 µg/ml) for 24 h to induce KMO expression or left untreated. RNA was isolated and the effect of rs2275163 genotype on KMO pre-mRNA stability was examined using ddPCR with rs2275163 C/T-specific MGB probes (n=3). NTC = no template control; gDNA = THP-1 genomic DNA.

**
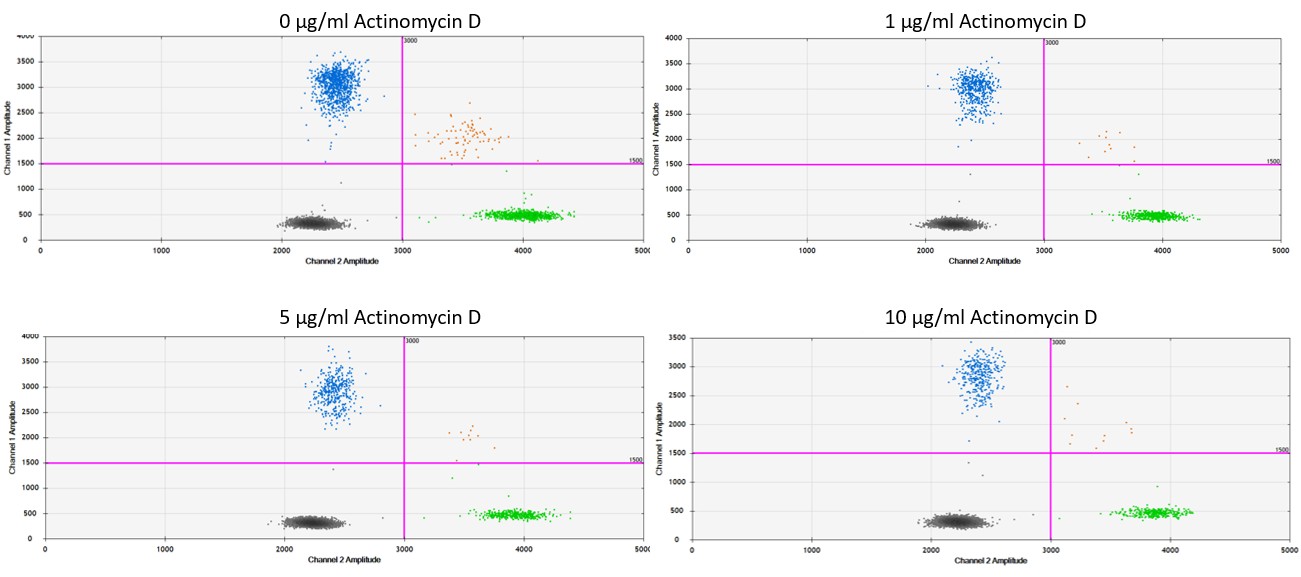
**

A

**
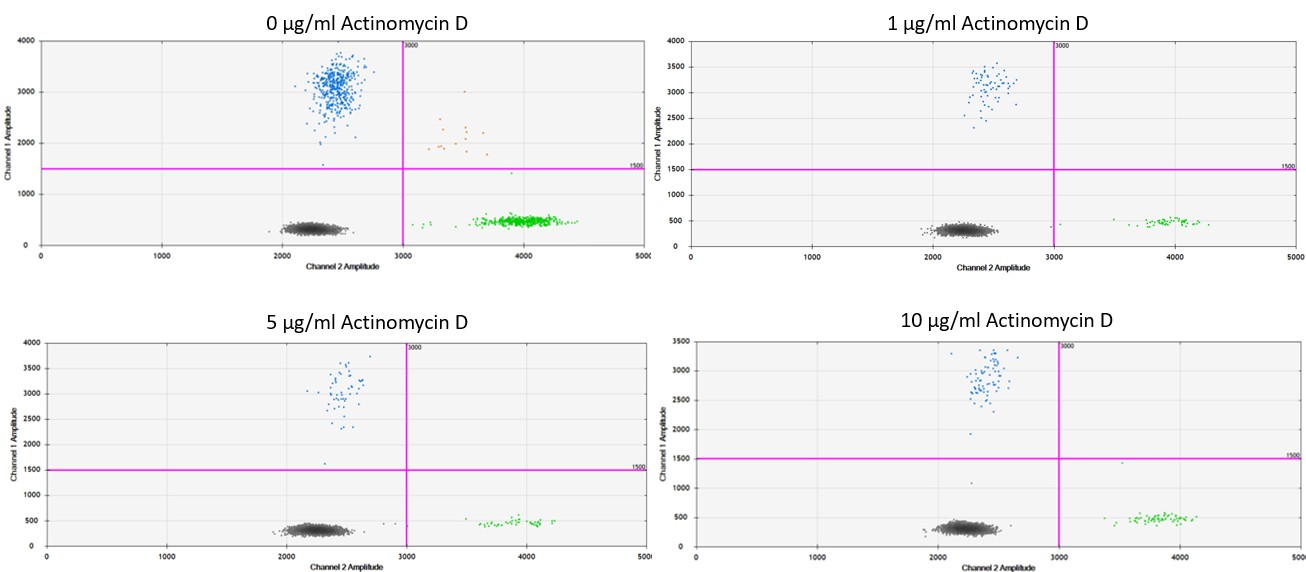
**

B

**Figure S2.** ddPCR dot plots of KMO pre-mRNA expression in THP-1 cells. THP-1 cells were treated with LPS (0.1 µg/ml) for 24 h to induce KMO expression. Cells were then treated with 1-10 µg/ml actinomycin D for 1 h (A) or 4 h (B). ddPCR with rs2275163 C/T-specific MGB probes was carried out on cDNA for each sample. Blue dots = C-FAM positive droplets; Green dots = T-HEX positive droplets; Orange dots = C+T positive droplets; Black dots = negative (empty) droplets.


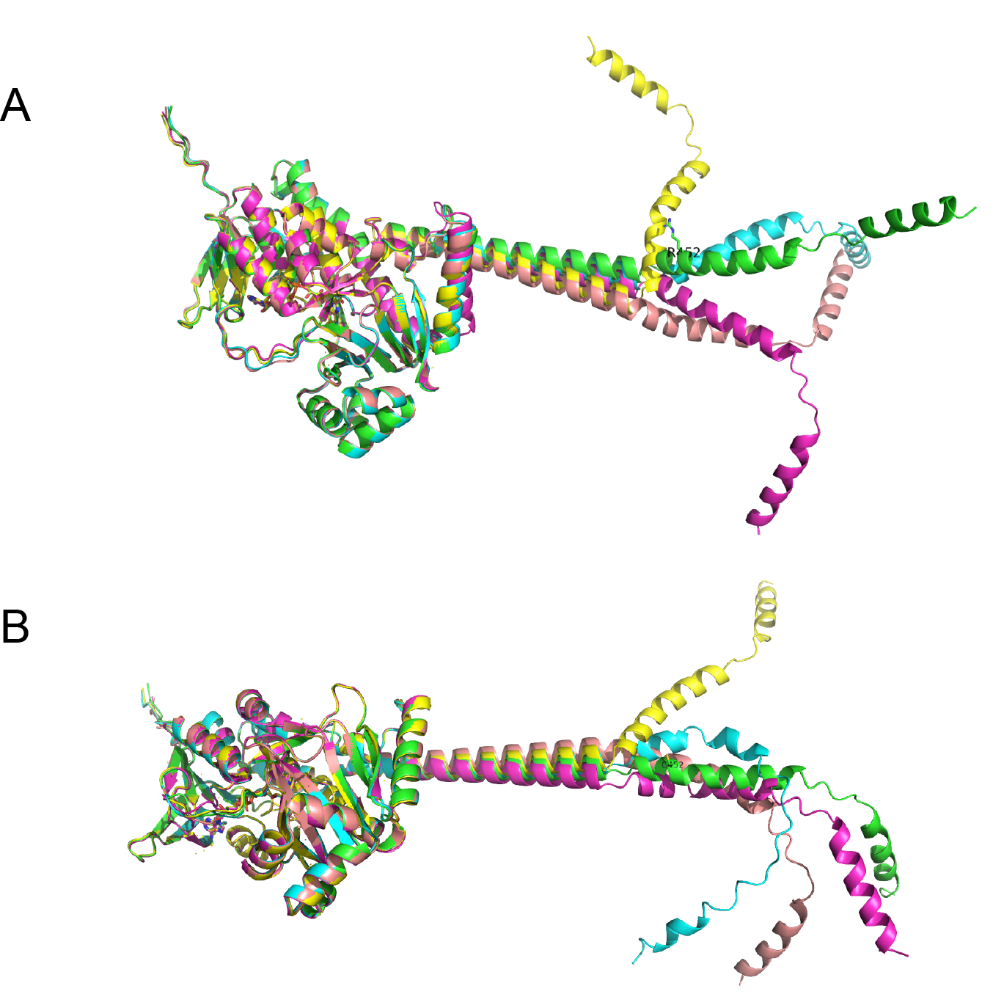


**Figure S3.** AlphaFold KMO variant structure prediction results. (A) The top 5 structures predicted from AlphaFold3 for variant Arg452. (B) The top 5 structures predicted from AlphaFold3 for variant Cys452.


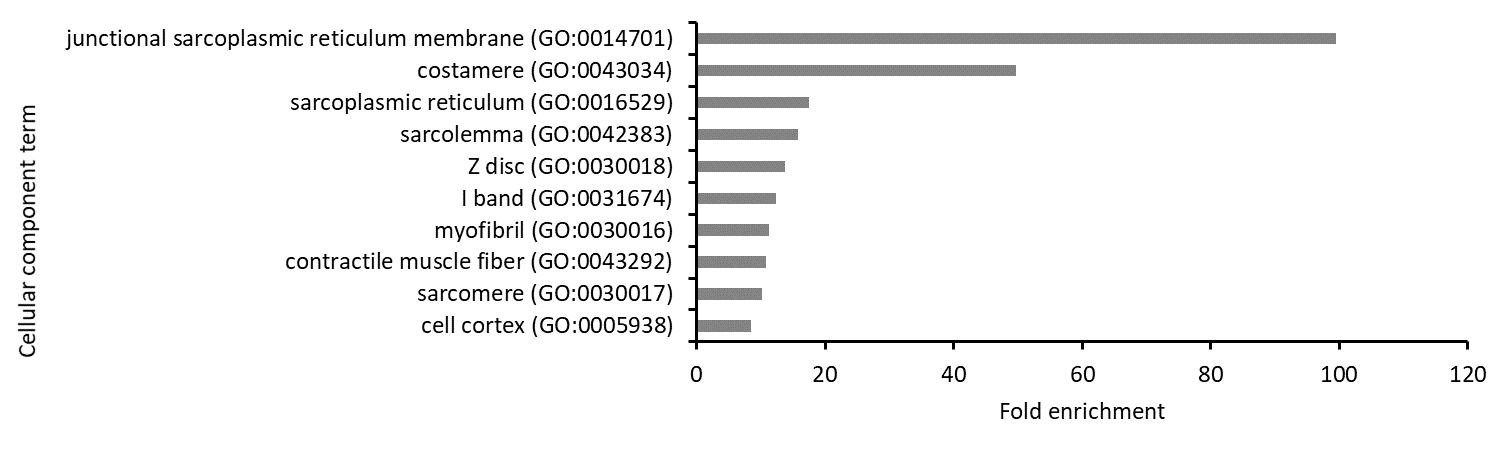

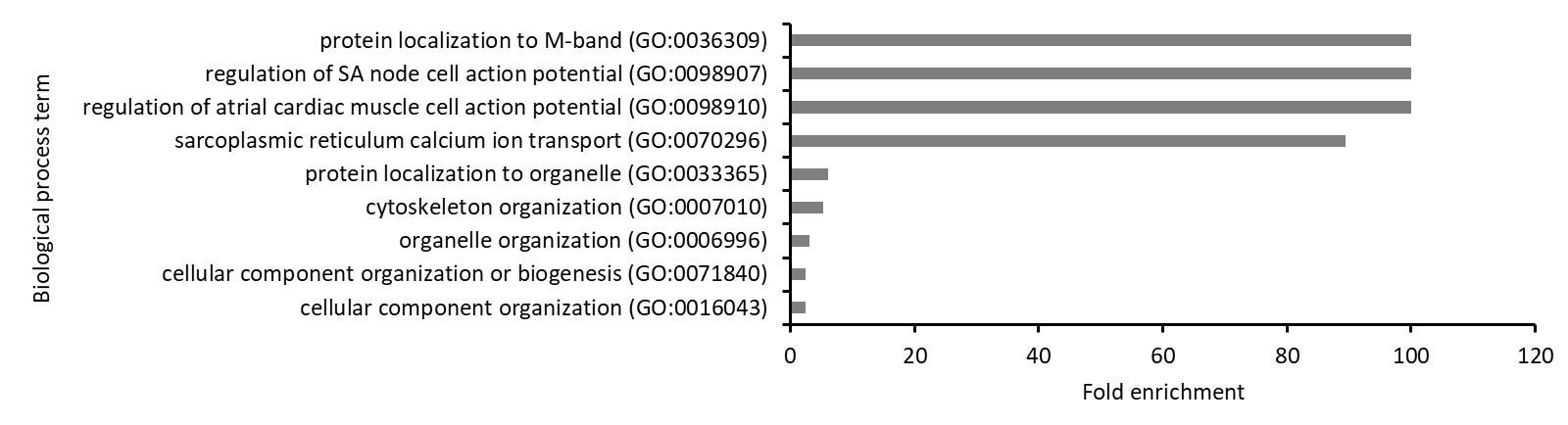


A

B


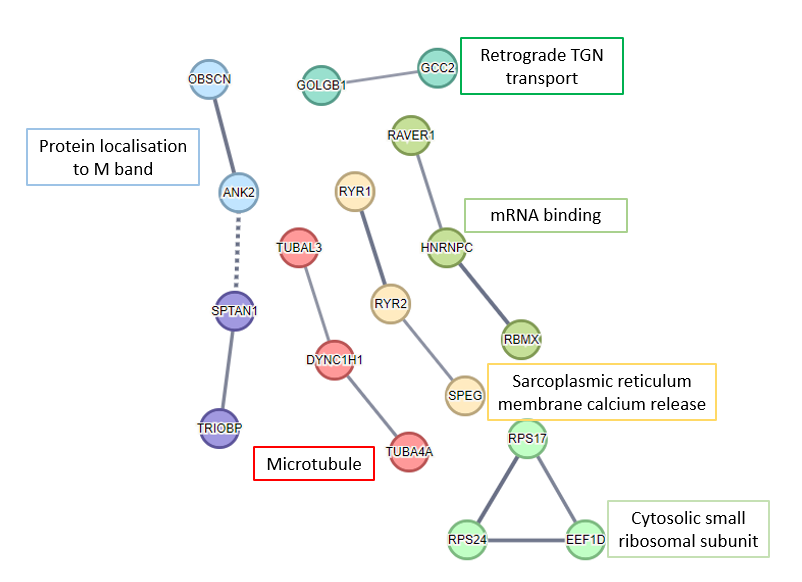


C

**Figure S4.** Gene ontology and STRING protein interaction network analysis of KMO-interacting proteins shared between KMO-Arg452 and KMO-Cys452 with confidence scores ≥40 (top ten GO terms are shown). A) GO-term enrichment analysis to derive cellular component terms associated with KMO-Arg/Cys452 interacting proteins, B) biological process terms associated with KMO-Arg/Cys452 interacting proteins, and C) STRING protein interaction network analysis to derive networks associated with KMO-Arg/Cys452 interacting proteins. Network edges show confidence where the thickness of the lines represent the strength of the supporting data.

**Table S1.** Lists of proteins with confidence scores ≥40 that specifically interacted with the KMO-Arg452 or KMO-Cys452 variants.

| **Arg452** | **Cys452** |
| --- | --- |
| HSP90AA1 | ACTA1 |
| CLASP1 | DNAH14 |
| IPO5 | HDLBP |
| HNRNPK | PLEC |
| TUBA3E | SPTBN4 |
| ZC3HAV1 | HDAC6 |
| RBBP7 | DSP |
| RPL17 | GAK |
| FSIP2 | CLASP2 |
| OTOF | EPPK1 |
| SPEN | PI4KA |
| ALDOA | CCDC88B |
| MYO9A | ATP1A2 |
| PTBP3 | ZC3H13 |
| NUMA1 | CEP135 |
| CENPE | NIN |
| MYH11 | CCDC144A |
| VAPA | AP3D1 |
| DUOX1 | CKAP5 |
| UPF2 | RPN1 |
| MTAP | SH3GLB1 |
| RYR3 | RIF1 |
| H3F3C | PPFIA2 |
| HMCN1 | TPM3 |
| SACS | PDZD2 |
| SPTBN1 | NWD1 |
| MAP1A | EIF4G3 |
| RAB15 | DARS |
| RPL28 | KRT13 |
| KIF16B | LRRFIP1 |
| PPP1R12A | CAPN1 |
| ARF3 | RPL36AL |
| ARID4A | DDX4 |
| SMG1 | ABCA10 |
| JPH2 | RBBP6 |
| MYH14 | SRP72 |
| C10orf112 | YTHDF3 |
| TIMM50 | ATP2A2 |
| PLEKHG5 | CCDC66 |
| KIF21A | HNRNPA3 |
| KIF6 | DGKA |
| GVINP1 | LIMCH1 |
| CLPB | RGPD1 |
| SOGA1 | SEPT9 |
| LRP2 | PLB1 |
| DOCK4 | CYFIP1 |
| CELSR3 | HMGB1 |
| YLPM1 | UBR4 |
| MT1G | TSC1 |
| CCDC73 | CDK12 |
| REV1 | KIAA0100 |
| SART3 | SYNJ1 |
| RABEP1 | ANKRD62 |
| GLOD4 | RGPD3 |
| ZNF831 | AFAP1L1 |
| ATRX | CCDC30 |
| PSME4 | DCTN1 |
| PPP2R2B | RNF113A |
| ACSL4 | ZNF791 |
| KIAA1551 | STAU1 |
| DHX8 | ADAM22 |
| CAMSAP2 | BCOR |
| AMPD2 | CLIP4 |
|  | TMPO |
|  | GNB2 |
|  | ZNFX1 |
|  | RPRD2 |
|  | SIPA1L3 |
|  | SVIL |
|  | IFT88 |
|  | CDC5L |
|  | WNK1 |
|  | LAMA4 |
|  | RRM2 |
|  | L1TD1 |
|  | CHD6 |
|  | ABCA13 |
|  | MAPK10 |
|  | BTN3A1 |
|  | ROCK1 |
|  | AK9 |
|  | MITF |
|  | CNTRL |
|  | RFC4 |
|  | CCDC81 |
|  | H2AFZ |
|  | GNAI3 |
|  | DOCK5 |
|  | USP1 |
|  | CDKN2A |
|  | LRRK2 |
|  | NHSL1 |
|  | EP400 |
|  | GRID2IP |
|  | CENPF |
|  | AXDND1 |
|  | ZNF571 |
|  | WDR33 |
|  | MATR3 |
|  | GRK7 |
|  | USP47 |
|  | CAPRIN2 |
|  | MYO10 |
|  | PPFIA3 |
|  | SLFN13 |
|  | PROSC |
|  | KCTD16 |

**Table S2.** GO cellular component analysis of KMO-Arg452 interacting proteins

| **GO cellular component terms** | **Number of genes in *Homo sapiens* reference list** | **Number of genes in uploaded data set that map to reference list** | **Number of genes in uploaded data set expected to map to reference list** | **Fold Enrichment of genes in data set compared to the expected number of genes** | **Raw P-value** | **False Discovery Rate** |
| --- | --- | --- | --- | --- | --- | --- |
| cortical microtubule cytoskeleton (GO:0030981) | 3 | 2 | 0.01 | 100 | 2.59E-05 | 4.68E-03 |
| microtubule minus-end (GO:0036449) | 7 | 2 | 0.02 | 96.39 | 1.80E-04 | 1.63E-02 |
| mitotic spindle midzone (GO:1990023) | 14 | 2 | 0.04 | 48.2 | 7.69E-04 | 4.63E-02 |
| microtubule end (GO:1990752) | 34 | 3 | 0.1 | 29.77 | 1.39E-04 | 1.32E-02 |
| A band (GO:0031672) | 40 | 3 | 0.12 | 25.3 | 2.26E-04 | 1.80E-02 |
| kinesin complex (GO:0005871) | 48 | 3 | 0.14 | 21.09 | 3.90E-04 | 2.98E-02 |
| myosin complex (GO:0016459) | 58 | 3 | 0.17 | 17.45 | 6.81E-04 | 4.37E-02 |
| contractile muscle fiber (GO:0043292) | 249 | 6 | 0.74 | 8.13 | 9.40E-05 | 1.25E-02 |
| sarcomere (GO:0030017) | 218 | 5 | 0.65 | 7.74 | 4.68E-04 | 3.32E-02 |
| myofibril (GO:0030016) | 239 | 5 | 0.71 | 7.06 | 7.10E-04 | 4.41E-02 |
| microtubule (GO:0005874) | 483 | 10 | 1.43 | 6.99 | 1.43E-06 | 7.09E-04 |
| supramolecular fiber (GO:0099512) | 1063 | 18 | 3.15 | 5.71 | 9.43E-10 | 9.38E-07 |
| supramolecular polymer (GO:0099081) | 1071 | 18 | 3.17 | 5.67 | 1.06E-09 | 7.05E-07 |
| supramolecular complex (GO:0099080) | 1439 | 22 | 4.27 | 5.16 | 5.11E-11 | 1.02E-07 |
| axon (GO:0030424) | 651 | 9 | 1.93 | 4.66 | 1.20E-04 | 1.25E-02 |
| polymeric cytoskeletal fiber (GO:0099513) | 818 | 11 | 2.42 | 4.54 | 2.48E-05 | 4.94E-03 |
| microtubule cytoskeleton (GO:0015630) | 1419 | 13 | 4.21 | 3.09 | 2.19E-04 | 1.82E-02 |
| neuron projection (GO:0043005) | 1336 | 12 | 3.96 | 3.03 | 4.76E-04 | 3.27E-02 |
| cytoskeleton (GO:0005856) | 2444 | 20 | 7.24 | 2.76 | 1.41E-05 | 3.12E-03 |
| extracellular exosome (GO:0070062) | 2102 | 17 | 6.23 | 2.73 | 8.95E-05 | 1.27E-02 |
| extracellular vesicle (GO:1903561) | 2131 | 17 | 6.32 | 2.69 | 1.06E-04 | 1.32E-02 |
| extracellular organelle (GO:0043230) | 2132 | 17 | 6.32 | 2.69 | 1.07E-04 | 1.25E-02 |
| extracellular membrane-bounded organelle (GO:0065010) | 2132 | 17 | 6.32 | 2.69 | 1.07E-04 | 1.18E-02 |
| cell projection (GO:0042995) | 2405 | 17 | 7.13 | 2.38 | 4.56E-04 | 3.36E-02 |
| intracellular non-membrane-bounded organelle (GO:0043232) | 5359 | 32 | 15.88 | 2.01 | 1.21E-05 | 3.43E-03 |
| non-membrane-bounded organelle (GO:0043228) | 5361 | 32 | 15.89 | 2.01 | 1.21E-05 | 3.02E-03 |
| cytosol (GO:0005829) | 5513 | 31 | 16.34 | 1.9 | 6.52E-05 | 1.08E-02 |
| intracellular organelle (GO:0043229) | 13324 | 55 | 39.49 | 1.39 | 9.96E-06 | 3.30E-03 |
| cytoplasm (GO:0005737) | 12190 | 50 | 36.13 | 1.38 | 2.07E-04 | 1.79E-02 |
| organelle (GO:0043226) | 14124 | 57 | 41.86 | 1.36 | 5.00E-06 | 1.99E-03 |
| intracellular membrane-bounded organelle (GO:0043231) | 12180 | 49 | 36.1 | 1.36 | 5.99E-04 | 3.97E-02 |
| membrane-bounded organelle (GO:0043227) | 13258 | 53 | 39.3 | 1.35 | 1.32E-04 | 1.31E-02 |
| intracellular anatomical structure (GO:0005622) | 14978 | 57 | 44.4 | 1.28 | 7.20E-05 | 1.10E-02 |

**Table S3**. KMO-Arg452-interacting proteins associated with GO terms of interest

| **cortical microtubule cytoskeleton (GO:0030981)** |  |
| --- | --- |
| **Gene name** | **Protein name** |
| NUMA1 | Nuclear mitotic apparatus protein 1 |
| CLASP1 | Isoform 3 of CLIP-associating protein 1 |
|  |  |
| **kinesin complex (GO:0005871)** |  |
| **Gene name** | **Protein name** |
| KIF6 | Kinesin-like protein KIF6 |
| KIF16B | Kinesin-like protein KIF16B |
| KIF21A | Kinesin-like protein KIF21A |
|  |  |
| **myosin complex (GO:0016459)** |  |
| **Gene name** | **Protein name** |
| MYO9A | Myosin XIA |
| MYH11 | Myosin heavy chain 11 |
| MYH14 | Myosin heavy chain 14 |
|  |  |
| **contractile muscle fiber (GO:0043292)** |  |
| **Gene name** | **Protein name** |
| MYH11 | Myosin heavy chain 11 |
| ALDOA | Isoform 2 of Fructose-bisphosphate aldolase A |
| RYR3 | Ryanodine receptor 3 |
| SPTBN1 | Spectrin beta chain_ non-erythrocytic 1 |
| PPP1R12A | Protein phosphatase 1 regulatory subunit 12A |
| JPH2 | Junctophilin 2 |
|  |  |
| **microtubule end (GO:1990752)** |  |
| **Gene name** | **Protein name** |
| CAMSAP2 | Calmodulin-regulated spectrin-associated protein 2 |
| NUMA1 | Nuclear mitotic apparatus protein 1 |
| CLASP1 | Isoform 3 of CLIP-associating protein 1 |
|  |  |
| **mitotic spindle midzone (GO:1990023)** |  |
| **Gene name** | **Protein name** |
| CENPE | Centromere protein E |
| NUMA1 | Nuclear mitotic apparatus protein 1 |

**Table S4.** GO biological process (A) and cellular component (B) analysis of KMO-Cys452 interacting proteins

| 1. **GO biological process terms** | **Number of genes in *Homo sapiens* reference list** | **Number of genes in uploaded data set that map to reference list** | **Number of genes in uploaded data set expected to map to reference list** | **Fold Enrichment of genes in data set compared to the expected number of genes** | **Raw P-value** | **False Discovery Rate** |
| --- | --- | --- | --- | --- | --- | --- |
| centriole-centriole cohesion (GO:0010457) | 13 | 3 | 0.07 | 43.97 | 3.87E-05 | 3.90E-02 |
| regulation of synaptic vesicle recycling (GO:1903421) | 26 | 4 | 0.14 | 29.32 | 9.81E-06 | 2.97E-02 |
| synapse organization (GO:0050808) | 337 | 10 | 1.77 | 5.65 | 1.14E-05 | 2.45E-02 |
| regulation of autophagy (GO:0010506) | 363 | 10 | 1.9 | 5.25 | 2.15E-05 | 3.62E-02 |
| regulation of actin filament-based process (GO:0032970) | 382 | 10 | 2 | 4.99 | 3.32E-05 | 4.19E-02 |
| regulation of supramolecular fiber organization (GO:1902903) | 387 | 10 | 2.03 | 4.92 | 3.71E-05 | 4.01E-02 |
| supramolecular fiber organization (GO:0097435) | 596 | 14 | 3.13 | 4.48 | 2.87E-06 | 4.34E-02 |
| cell junction organization (GO:0034330) | 533 | 12 | 2.8 | 4.29 | 2.35E-05 | 3.23E-02 |
| regulation of cellular component biogenesis (GO:0044087) | 979 | 18 | 5.14 | 3.5 | 3.23E-06 | 2.44E-02 |
| positive regulation of cellular component organization (GO:0051130) | 1115 | 18 | 5.85 | 3.08 | 1.91E-05 | 3.61E-02 |
| cytoskeleton organization (GO:0007010) | 1256 | 20 | 6.59 | 3.03 | 7.50E-06 | 2.83E-02 |
| cellular response to stress (GO:0033554) | 1589 | 22 | 8.34 | 2.64 | 2.22E-05 | 3.36E-02 |
| organelle organization (GO:0006996) | 3099 | 35 | 16.26 | 2.15 | 5.05E-06 | 2.55E-02 |
| cellular component biogenesis (GO:0044085) | 2705 | 30 | 14.2 | 2.11 | 4.50E-05 | 4.25E-02 |
| cellular component organization (GO:0016043) | 5623 | 51 | 29.51 | 1.73 | 1.07E-05 | 2.70E-02 |
| cellular component organization or biogenesis (GO:0071840) | 5838 | 51 | 30.64 | 1.66 | 3.66E-05 | 4.25E-02 |

| 1. **GO cellular component terms** | **Number of genes in *Homo sapiens* reference list** | **Number of genes in uploaded data set that map to reference list** | **Number of genes in uploaded data set expected to map to reference list** | **Fold Enrichment of genes in data set compared to the expected number of genes** | **Raw P-value** | **False Discovery Rate** |
| --- | --- | --- | --- | --- | --- | --- |
| centriolar subdistal appendage (GO:0120103) | 10 | 3 | 0.05 | 57.17 | 1.64E-05 | 1.48E-03 |
| hemidesmosome (GO:0030056) | 7 | 2 | 0.04 | 54.44 | 5.63E-04 | 3.20E-02 |
| ciliary transition fiber (GO:0097539) | 8 | 2 | 0.04 | 47.64 | 7.48E-04 | 3.92E-02 |
| microtubule plus-end (GO:0035371) | 24 | 3 | 0.13 | 23.82 | 2.63E-04 | 1.63E-02 |
| microtubule end (GO:1990752) | 34 | 4 | 0.18 | 22.42 | 2.95E-05 | 2.44E-03 |
| podosome (GO:0002102) | 33 | 3 | 0.17 | 17.32 | 6.84E-04 | 3.68E-02 |
| terminal bouton (GO:0043195) | 46 | 4 | 0.24 | 16.57 | 9.88E-05 | 7.02E-03 |
| cell cortex region (GO:0099738) | 35 | 3 | 0.18 | 16.33 | 8.14E-04 | 4.05E-02 |
| contractile actin filament bundle (GO:0097517) | 85 | 6 | 0.45 | 13.45 | 5.67E-06 | 7.05E-04 |
| stress fiber (GO:0001725) | 85 | 6 | 0.45 | 13.45 | 5.67E-06 | 6.64E-04 |
| actomyosin (GO:0042641) | 91 | 6 | 0.48 | 12.56 | 8.43E-06 | 8.82E-04 |
| actin filament bundle (GO:0032432) | 94 | 6 | 0.49 | 12.16 | 1.02E-05 | 1.01E-03 |
| cytoplasmic region (GO:0099568) | 308 | 9 | 1.62 | 5.57 | 3.58E-05 | 2.85E-03 |
| midbody (GO:0030496) | 206 | 6 | 1.08 | 5.55 | 7.67E-04 | 3.91E-02 |
| cell cortex (GO:0005938) | 314 | 8 | 1.65 | 4.85 | 2.52E-04 | 1.62E-02 |
| polymeric cytoskeletal fiber (GO:0099513) | 818 | 19 | 4.29 | 4.43 | 4.66E-08 | 1.85E-05 |
| centrosome (GO:0005813) | 737 | 17 | 3.87 | 4.4 | 2.86E-07 | 8.12E-05 |
| microtubule (GO:0005874) | 483 | 11 | 2.53 | 4.34 | 4.72E-05 | 3.61E-03 |
| actin cytoskeleton (GO:0015629) | 521 | 11 | 2.73 | 4.02 | 9.29E-05 | 6.84E-03 |
| cell-substrate junction (GO:0030055) | 434 | 9 | 2.28 | 3.95 | 4.71E-04 | 2.75E-02 |
| microtubule organizing center (GO:0005815) | 879 | 18 | 4.61 | 3.9 | 6.99E-07 | 1.39E-04 |
| microtubule cytoskeleton (GO:0015630) | 1419 | 26 | 7.45 | 3.49 | 1.42E-08 | 7.04E-06 |
| supramolecular fiber (GO:0099512) | 1063 | 19 | 5.58 | 3.41 | 2.51E-06 | 3.56E-04 |
| supramolecular polymer (GO:0099081) | 1071 | 19 | 5.62 | 3.38 | 2.80E-06 | 3.71E-04 |
| anchoring junction (GO:0070161) | 902 | 16 | 4.73 | 3.38 | 1.88E-05 | 1.63E-03 |
| supramolecular complex (GO:0099080) | 1439 | 25 | 7.55 | 3.31 | 8.07E-08 | 2.68E-05 |
| axon (GO:0030424) | 651 | 11 | 3.42 | 3.22 | 6.25E-04 | 3.45E-02 |
| cytoskeleton (GO:0005856) | 2444 | 41 | 12.83 | 3.2 | 2.77E-12 | 5.52E-09 |
| cell junction (GO:0030054) | 2238 | 30 | 11.74 | 2.55 | 9.06E-07 | 1.50E-04 |
| cell projection (GO:0042995) | 2405 | 31 | 12.62 | 2.46 | 1.31E-06 | 2.00E-04 |
| plasma membrane bounded cell projection (GO:0120025) | 2291 | 26 | 12.02 | 2.16 | 1.48E-04 | 9.80E-03 |
| intracellular non-membrane-bounded organelle (GO:0043232) | 5359 | 57 | 28.12 | 2.03 | 3.40E-09 | 3.38E-06 |
| non-membrane-bounded organelle (GO:0043228) | 5361 | 57 | 28.13 | 2.03 | 3.44E-09 | 2.28E-06 |
| cytosol (GO:0005829) | 5513 | 54 | 28.93 | 1.87 | 2.89E-07 | 7.19E-05 |
| protein-containing complex (GO:0032991) | 6512 | 56 | 34.17 | 1.64 | 1.56E-05 | 1.48E-03 |
| intracellular organelle (GO:0043229) | 13324 | 93 | 69.92 | 1.33 | 8.14E-07 | 1.47E-04 |
| organelle (GO:0043226) | 14124 | 97 | 74.12 | 1.31 | 3.32E-07 | 7.35E-05 |
| cytoplasm (GO:0005737) | 12190 | 82 | 63.97 | 1.28 | 3.63E-04 | 2.19E-02 |
| membrane-bounded organelle (GO:0043227) | 13258 | 88 | 69.58 | 1.26 | 1.16E-04 | 7.95E-03 |
| intracellular anatomical structure (GO:0005622) | 14978 | 98 | 78.6 | 1.25 | 6.07E-06 | 6.71E-04 |

**Table S5**. KMO-Cys452-interacting proteins associated with GO terms of interest

| **centriole-centriole cohesion (GO:0010457)** |  |
| --- | --- |
| **Gene name** | **Protein name** |
| NIN | Isoform 3 of Ninein |
| CEP135 | Centrosomal protein 135 |
| DCTN1 | Dynactin subunit 1 |
|  |  |
| **regulation of synaptic vesicle recycling (GO:1903421)** |  |
| **Gene name** | **Protein name** |
| CYFIP1 | Cytoplasmic FMR1-interacting protein 1 |
| ABCA13 | ATP binding cassette subfamily A member 13 |
| ROCK1 | Rho-associated protein kinase 1 |
| LRRK2 | Leucine-rich repeat serine/threonine-protein kinase 2 |
|  |  |
| **synapse organization (GO:0050808)** |  |
| **Gene name** | **Protein name** |
| SPTBN4 | Spectrin beta, non-erythrocytic 4 |
| CLASP2 | CLIP-associating protein 2 |
| PPFIA2 | PTPRF interacting protein alpha 2/ Liprin alpha 2 |
| TSC1 | TSC complex subunit 1 |
| CYFIP1 | Cytoplasmic FMR1-interacting protein 1 |
| STAU1 | Staufen double-stranded RNA binding protein 1 |
| LRRK2 | Leucine-rich repeat serine/threonine-protein kinase 2 |
| PPFIA3 | PTPRF interacting protein alpha 3 |
| DCTN1 | Dynactin subunit 1 |
| HDAC6 | Isoform 2 of Histone deacetylase 6 |
|  |  |
| **terminal bouton (GO:0043195)** |  |
| **Gene name** | **Protein name** |
| AP3D1 | AP-3 complex subunit delta-1 |
| CYFIP1 | Cytoplasmic FMR1-interacting protein 1 |
| SYNJ1 | Synaptojanin-1 |
| LRRK2 | Leucine-rich repeat serine/threonine-protein kinase 2 |
|  |  |
| **regulation of autophagy (GO:0010506)** |  |
| **Gene name** | **Protein name** |
| UBR4 | Ubiquitin protein ligase E3 component n-recognin 4 |
| CAPN1 | Calpain 1 |
| TSC1 | TSC complex subunit 1 |
| WNK1 | WNK lysine deficient protein kinase 1 |
| ROCK1 | Rho-associated protein kinase 1 |
| LRRK2 | Leucine-rich repeat serine/threonine-protein kinase 2 |
| HDAC6 | Isoform 2 of Histone deacetylase 6 |
| SH3GLB1 | SH3 domain containing GRB2 like, endophilin B1 |
| HMGB1 | High mobility group box 1 |
| GNAI3 | G protein subunit alpha i3 |
|  |  |
| **supramolecular fiber organization (GO:0097435)** |  |
| **Gene name** | **Protein name** |
| PLEC | Isoform 2 of Plectin |
| CCDC88B | Coiled-coil domain containing 88B |
| CKAP5 | Cytoskeleton-associated protein 5 |
| CLASP2 | CLIP-associating protein 2 |
| DSP | Desomoplakin |
| NIN | Isoform 3 of Ninein |
| EPPK1 | Epiplakin 1 |
| TPM3 | Isoform 2 of Tropomyosin alpha-3 chain |
| CLIP4 | CAP-Gly domain-containing linker protein 4 |
| KRT13 | Keratin_ type I cytoskeletal 13 |
| ACTA1 | Actin_ alpha skeletal muscle |
| SVIL | Supervillin |
| HDAC6 | Isoform 2 of Histone deacetylase 6 |
| CDKN2A | Cyclin dependent kinase inhibitor 2A |
|  |  |
| **microtubule plus-end (GO:0035371)** |  |
| **Gene name** | **Protein name** |
| CKAP5 | Cytoskeleton-associated protein 5 |
| DCTN1 | Dynactin subunit 1 |
| CLIP4 | CAP-Gly domain-containing linker protein 4 |
|  |  |
| **centriolar subdistal appendage (GO:0120103)** |  |
| **Gene name** | **Protein name** |
| NIN | Isoform 3 of Ninein |
| CNTRL | Centriolin |
| DCTN1 | Dynactin subunit 1 |
|  |  |
| **contractile actin filament bundle (GO:0097517)** |  |
| **Gene name** | **Protein name** |
| TPM3 | Isoform 2 of Tropomyosin alpha-3 chain |
| LIMCH1 | LIM and calponin homology domains 1 |
| SIPA1L3 | Signal induced proliferation associated 1 like 3 |
| AFAP1L1 | Actin filament associated protein 1 like 1 |
| ACTA1 | Actin_ alpha skeletal muscle |
| SEPT9 | Septin 9 |
|  |  |

**Table S6.** Proteins with confidence scores ≥40 for both KMO variants and that interacted with both KMO-Arg452 and KMO-Cys452.

| **Both Arg452 and Cys452** |
| --- |
| SYNE1 |
| KMO |
| DYNC1H1 |
| TUBA4A |
| AHNAK |
| GOLGB1 |
| OBSCN |
| FAM179B |
| MYH10 |
| RBMX |
| RAVER1 |
| GSR |
| TTK |
| RYR2 |
| KRT17 |
| LARP1B |
| BRWD1 |
| MAP4 |
| RPLP0P6 |
| RCN2 |
| CEP290 |
| PABPC1L |
| LDHA |
| GCC2 |
| ATP1A3 |
| BAX |
| SPTAN1 |
| BAG6 |
| ERC1 |
| RPS24 |
| SUPT6H |
| TUBAL3 |
| RGPD5 |
| SAMHD1 |
| EEF1D |
| TRIOBP |
| RPS17 |
| RYR1 |
| ANK2 |
| HELLS |
| HNRNPC |
| EIF4B |
| FGD2 |
| HAX1 |
| SPEG |
| GTF2IRD2 |

**Table S7.** GO biological process (A) and cellular component (B) analysis of KMO-Arg/Cys452 interacting proteins

| **A) GO biological process terms** | **Number of genes in *Homo sapiens* reference list** | **Number of genes in uploaded data set that map to reference list** | **Number of genes in uploaded data set expected to map to reference list** | **Fold Enrichment of genes in data set compared to the expected number of genes** | **Raw P-value** | **False Discovery Rate** |
| --- | --- | --- | --- | --- | --- | --- |
| protein localization to M-band (GO:0036309) | 2 | 2 | 0 | 100 | 4.89E-06 | 1.06E-02 |
| regulation of SA node cell action potential (GO:0098907) | 3 | 2 | 0.01 | 100 | 1.46E-05 | 2.77E-02 |
| regulation of atrial cardiac muscle cell action potential (GO:0098910) | 4 | 2 | 0.01 | 100 | 2.92E-05 | 4.91E-02 |
| sarcoplasmic reticulum calcium ion transport (GO:0070296) | 15 | 3 | 0.03 | 89.48 | 4.67E-06 | 1.18E-02 |
| protein localization to organelle (GO:0033365) | 739 | 10 | 1.65 | 6.05 | 4.24E-06 | 1.28E-02 |
| cytoskeleton organization (GO:0007010) | 1256 | 15 | 2.81 | 5.34 | 4.75E-08 | 3.59E-04 |
| organelle organization (GO:0006996) | 3099 | 21 | 6.93 | 3.03 | 7.64E-07 | 2.89E-03 |
| cellular component organization or biogenesis (GO:0071840) | 5838 | 32 | 13.05 | 2.45 | 7.98E-09 | 1.21E-04 |
| cellular component organization (GO:0016043) | 5623 | 30 | 12.57 | 2.39 | 8.92E-08 | 4.49E-04 |

| **B) GO cellular component terms** | **Number of genes in *Homo sapiens* reference list** | **Number of genes in uploaded data set that map to reference list** | **Number of genes in uploaded data set expected to map to reference list** | **Fold Enrichment of genes in data set compared to the expected number of genes** | **Raw P-value** | **False Discovery Rate** |
| --- | --- | --- | --- | --- | --- | --- |
| junctional sarcoplasmic reticulum membrane (GO:0014701) | 9 | 2 | 0.02 | 99.42 | 1.74E-04 | 1.65E-02 |
| costamere (GO:0043034) | 18 | 2 | 0.04 | 49.71 | 7.31E-04 | 4.69E-02 |
| sarcoplasmic reticulum (GO:0016529) | 77 | 3 | 0.17 | 17.43 | 6.81E-04 | 4.51E-02 |
| sarcolemma (GO:0042383) | 142 | 5 | 0.32 | 15.75 | 1.59E-05 | 2.64E-03 |
| Z disc (GO:0030018) | 130 | 4 | 0.29 | 13.77 | 2.02E-04 | 1.83E-02 |
| I band (GO:0031674) | 144 | 4 | 0.32 | 12.43 | 2.99E-04 | 2.47E-02 |
| myofibril (GO:0030016) | 239 | 6 | 0.53 | 11.23 | 1.46E-05 | 2.65E-03 |
| contractile muscle fiber (GO:0043292) | 249 | 6 | 0.56 | 10.78 | 1.84E-05 | 2.82E-03 |
| sarcomere (GO:0030017) | 218 | 5 | 0.49 | 10.26 | 1.23E-04 | 1.43E-02 |
| cell cortex (GO:0005938) | 314 | 6 | 0.7 | 8.55 | 6.74E-05 | 8.38E-03 |
| cytoplasmic region (GO:0099568) | 308 | 5 | 0.69 | 7.26 | 6.03E-04 | 4.28E-02 |
| supramolecular fiber (GO:0099512) | 1063 | 13 | 2.38 | 5.47 | 3.58E-07 | 1.42E-04 |
| supramolecular polymer (GO:0099081) | 1071 | 13 | 2.39 | 5.43 | 3.90E-07 | 1.29E-04 |
| supramolecular complex (GO:0099080) | 1439 | 17 | 3.22 | 5.29 | 5.18E-09 | 3.44E-06 |
| cytoskeleton (GO:0005856) | 2444 | 19 | 5.46 | 3.48 | 4.19E-07 | 1.19E-04 |
| extracellular vesicle (GO:1903561) | 2131 | 14 | 4.76 | 2.94 | 1.52E-04 | 1.68E-02 |
| extracellular organelle (GO:0043230) | 2132 | 14 | 4.77 | 2.94 | 1.53E-04 | 1.60E-02 |
| extracellular membrane-bounded organelle (GO:0065010) | 2132 | 14 | 4.77 | 2.94 | 1.53E-04 | 1.52E-02 |
| extracellular exosome (GO:0070062) | 2102 | 13 | 4.7 | 2.77 | 5.08E-04 | 4.05E-02 |
| intracellular non-membrane-bounded organelle (GO:0043232) | 5359 | 32 | 11.98 | 2.67 | 7.83E-10 | 1.56E-06 |
| non-membrane-bounded organelle (GO:0043228) | 5361 | 32 | 11.98 | 2.67 | 7.91E-10 | 7.87E-07 |
| cell projection (GO:0042995) | 2405 | 14 | 5.38 | 2.6 | 5.39E-04 | 4.13E-02 |
| cytosol (GO:0005829) | 5513 | 28 | 12.32 | 2.27 | 1.81E-06 | 3.60E-04 |
| protein-containing complex (GO:0032991) | 6512 | 26 | 14.56 | 1.79 | 6.45E-04 | 4.43E-02 |
| nucleus (GO:0005634) | 7645 | 29 | 17.09 | 1.7 | 5.92E-04 | 4.36E-02 |
| intracellular organelle (GO:0043229) | 13324 | 45 | 29.78 | 1.51 | 7.08E-08 | 3.52E-05 |
| cytoplasm (GO:0005737) | 12190 | 40 | 27.25 | 1.47 | 6.28E-05 | 8.33E-03 |
| intracellular membrane-bounded organelle (GO:0043231) | 12180 | 39 | 27.22 | 1.43 | 2.46E-04 | 2.13E-02 |
| organelle (GO:0043226) | 14124 | 45 | 31.57 | 1.43 | 1.20E-06 | 2.65E-04 |
| membrane-bounded organelle (GO:0043227) | 13258 | 42 | 29.63 | 1.42 | 4.04E-05 | 5.74E-03 |
| intracellular anatomical structure (GO:0005622) | 14978 | 46 | 33.48 | 1.37 | 8.35E-07 | 2.08E-04 |

|  | **Controls (n=170)** | **SZ (n=156)** | **Test statistic** | **p-value** |
| --- | --- | --- | --- | --- |
| **Age (years)** | 37.6 ± 15.4 | 36.0 ± 13.5 | t=0.97 | .33 |
| **Sex (M/F)** | 94/76 | 104/52 | Χ^2^=4.41 | .036 |
| **rs1053230 genotype (CC/CT/TT)** | 137/33/0 | 111/40/5 | Χ^2^=7.81 | .020 |
| **Digit symbol coding task score** | 75.1 ± 17.6 | 59.1 ± 19.1 | t=7.76 | <.001 |
| **Digit sequencing task score** | 20.9 ± 4.4 | 17.5 ± 5.0 | t=6.30 | <.001 |
| **Antipsychotic type, n(%):**  **First generation**  **Second generation**  **Multiple**  **None** |  | 119 (76.3)  11 (7.1)  19 (12.2)  7 (4.5) |  |  |

**Table S8.** Characteristics of the cohort used for association of rs1053230 genotype with clinical parameters related to schizophrenia
